# Energy Landscapes for Base-Flipping in a Model DNA Duplex

**DOI:** 10.1021/acs.jpcb.2c00340

**Published:** 2022-04-15

**Authors:** Nicy, Debayan Chakraborty, David J. Wales

**Affiliations:** † Yusuf Hamied Department of Chemistry, University of Cambridge, Lensfield Road, Cambridge, CB2 1EW, U.K.; ‡ Department of Chemistry, The University of Texas at Austin, Austin, Texas 78712, United States

## Abstract

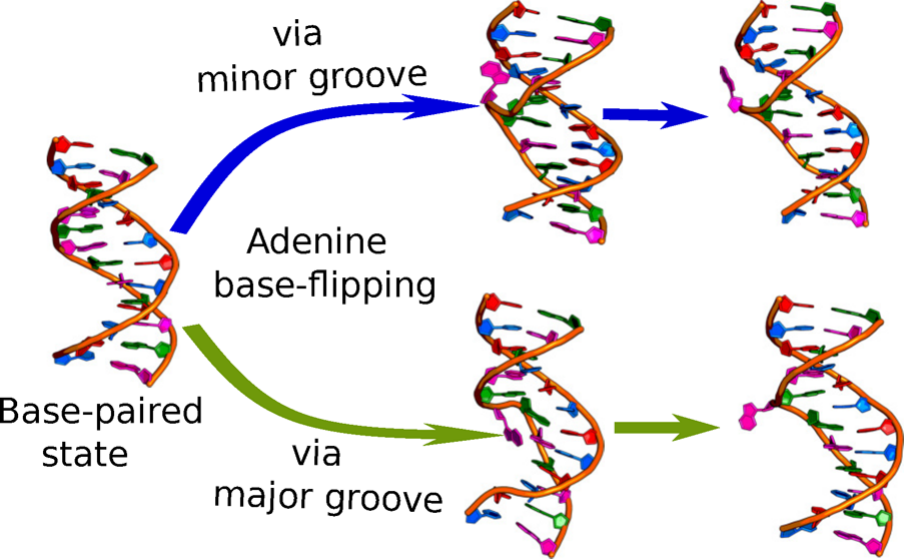

We explore the process of base-flipping for four central bases, adenine, guanine, cytosine, and thymine, in a deoxyribonucleic acid (DNA) duplex using the energy landscape perspective. NMR imino-proton exchange and fluorescence correlation spectroscopy studies have been used in previous experiments to obtain lifetimes for bases in paired and extrahelical states. However, the difference of almost 4 orders of magnitude in the base-flipping rates obtained by the two methods implies that they are exploring different pathways and possibly different open states. Our results support the previous suggestion that minor groove opening may be favored by distortions in the DNA backbone and reveal links between sequence effects and the direction of opening, i.e., whether the base flips toward the major or the minor groove side. In particular, base flipping along the minor groove pathway was found to align toward the 5′ side of the backbone. We find that bases align toward the 3′ side of the backbone when flipping along the major groove pathway. However, in some cases for cytosine and thymine, the base flipping along the major groove pathway also aligns toward the 5′ side. The sequence effect may be caused by the polar interactions between the flipping-base and its neighboring bases on either of the strands. For guanine flipping toward the minor groove side, we find that the equilibrium constant for opening is large compared to flipping via the major groove. We find that the estimated rates of base opening, and hence the lifetimes of the closed state, obtained for thymine flipping through small and large angles along the major groove differ by 6 orders of magnitude, whereas for thymine flipping through small angles along the minor groove and large angles along the major groove, the rates differ by 3 orders of magnitude.

## Introduction

The localized distortion within the DNA duplex, in which a single base breaks its hydrogen-bonding with the complementary base and unstacks out of the helix by rotating about 180° into an extrahelical position, is known as base-flipping.^1−3^ This process may occur spontaneously on a sufficiently long time scale (passive flipping), or an enzyme may be required to drive it (active flipping).^2^ Enzymes that are known to interact with a flipped-out base include methyltransferase (which can methylate cytosine (C) or adenine (A)),^4,5^ glycosylase (which removes thymine (T) or uracil (U) from a mismatched base-pair (bp)),^6−8^ endonuclease,^9^ integrase, helicase, polymerase, photolyase,^2,10^ and recombinase.^1^ These enzymes facilitate base-flipping out of the helix for several purposes. The first purpose is to access the genetic information contained within the duplex. The second purpose is to chemically modify the base, thereby influencing gene regulation. The most prominent example is base methylation, which plays an important role in epigenetics.^11^ The third purpose is to recognize and repair the damaged or chemically modified bases. Finally, the last one is to repair the mismatched base-pairs generated due to errors in copying by polymerases.^12−15^ Base-flipping may also play a role during transcription and replication.^16,17^

Defects in the working of enzymes associated with base-flipping are linked to several diseases. For example, the DNA repair machinery does not work efficiently in patients with xeroderma pigmentosum.^18^ Recently, defects in DNA glycosylases have been linked with colorectal cancer.^19,20^ Furthermore, by selectively hindering the repair pathway, it is possible to obtain improved antibiotics.^21^ For instance, hydrogen peroxide used in ion beam therapy for the treatment of cancer has been shown to stabilize base-pairs, making it difficult for enzymes to flip out the base during nucleotide excision repair (NER) and mismatch repair (MMR).^22^ Hence, inhibiting the DNA repair machinery can kill cancer cells and may thus provide a route to treatment.^23−25^

In 1925, Johnson and Coghill reported “The discovery of 5-Methyl-cytosine in tuberculinic acid, the nucleic acid of the tubercle bacillus”.^26^ However, as their identification was based solely on the optical properties of picrate, their report was met with scepticism.^27^ In 1948, the paper chromatography studies of Hotchkiss revealed the presence of epicytosine in thymus DNA.^28^ Finally, in 1950, equipped with Markham and Smith’s technique for detecting ultraviolet-absorbing molecules on a paper chromatogram, Wyatt demonstrated the existence of 5-methylcytosine in plant and animal nucleic acids.^27,29^ Since then, methylation of bases has been studied extensively, mainly for understanding how it influences gene regulation.^30^ It was during one such investigation in 1994 that Klimašauskas et al. first observed a cytosine base flipped outside a DNA helix. They detected this structure using X-ray crystallography for a ternary complex made up of *Hhal* DNA oligonucleotide, methyltransferase enzyme, and its cofactor.^4^ Earlier hydrogen-exchange kinetic studies using deuterium labeling and nuclear magnetic resonance (NMR) had already hinted at base-opening in nucleic acids.^31,32^

Base-flipping has since been investigated using a variety of experimental techniques, listed in Table 1. While the X-ray structure reported by Klimašauskas had the target base flipped out into the active site of an enzyme, several other cases have been subsequently reported.^3,4^ The enzyme may interact with the target base by flipping out its partner base,^3,33^ or it may flip both bases in the base-pair.^34,35^ Furthermore, the enzyme may cause a significant distortion of the sugar-phosphate backbone.^34,36^ Although X-ray crystallography successfully captures the static structure of the molecule at high resolution, it does not provide dynamic information.^3^

**Table 1:** Experimental Techniques Used for Studying Base-Flipping in a DNA Duplex

technique (advantage)	observation/prediction of lifetime of closed base-pair	limitations
X-ray	UDG follows major groove pathway^7^	- no information on dynamics^3^
(high resolution)	no lifetime information	- low solubility of large macromolecules^3^
		- different crystal and solution structures^3^
		
NMR imino-H	1–5 ms for AT bp^38^	- exact structure being monitored is not known
exchange studies	10–50 ms for GC bp^38^	- rate may be the rate of base wobbling^2,43−45,67^
(monitor dynamics)	91–122 ms for AT tracts^68^	- uncertain whether the target base,
	<5 ms for GC tracts^69^	or its partner base, or both, have flipped out^2^
		
FCS/ddFCS^52^	even in presence of enzymes,	- probe may not be specific to a base^2^
(monitor dynamics)	the lifetime obtained is of	- alteration of natural structure of DNA by^70^ insertion of probes
	the order of seconds.^46−50^	- indirect observation since probe is placed
	0.3–20 s for GT mismatched bp^53^	on base adjacent to the target base^71^
		
AFM	lifetime of closed bp even in	- results obtained depend on the interactions
(monitor dynamics	absence of stacking interactions	between the target molecule and the
at nm resolution)	is of the order of seconds^54^	surface it is attached to during AFM study^72^
		
host–guest	around 1000 s using	- difficult to obtain base-specific host^2^
complexation	β-cyclodextrin^58^	- macrocycle used to trap the flipped base may
(monitor dynamics)		induce base-flipping as in case of Bisacridine

NMR imino-proton (imino-H) exchange studies have been employed to monitor both spontaneous and enzymatic base-flipping in nucleic acids.^37−40^ This method is based on a two-state model, with the base either in a closed state or an open state. The imino-H on the N1 atom in guanine and the N3 atom in thymine can be exchanged when the base is in an open state.^38^ Interestingly, solid-state NMR and F-NMR (fluorine-NMR) studies have also explored the dynamics of base-flipping.^3,24,41,42^ Computational studies have revealed that the imino-H may be exchanged even when the base has moved slightly out of the helix.^2,43−45^ However, atomistic molecular dynamics simulations with standard force fields are not able to reproduce proton transfer reactions.

Several fluorescence correlation spectroscopy (FCS) studies have used 2-aminopurine and tetramethylrhodamine as fluorescent probes.^46−51^ However, it has not been possible to monitor spontaneous base-flipping effectively because of the time scale. In recent work using diffusion decelerated fluorescence correlation spectroscopy (ddFCS), Yin et al. determined the lifetime of a GT mismatched bp in a DNA duplex to be of the order of seconds.^52,53^ The lifetime of Watson–Crick (WC) base-pairs has been predicted to be longer than for mismatched base-pairs,^52,53^ and guanine–cytosine (GC) pairs are usually longer lived than adenine–thymine (AT) because of the third hydrogen-bond (H-bond).^38^

Base-flipping has also been analyzed using atomic force microscopy (AFM),^54−56^ host–guest complexation,^57−59^ total internal reflectance fluorescence microscopy (TIRFM),^60^ förster resonance energy transfer (FRET),^61^ photochemical approaches,^62^ and chemical probes.^63−66^

The fundamental question remains, how does the process of base-flipping occur at an atomic level of detail? What are the open states that are sampled during different experiments that make the flipping rates observed during NMR, and AFM, FCS and ddFCS^52^ differ by almost 4 orders of magnitude?^53^ How do enzymes recognize specific sequences of DNA, mismatched base-pairs, and chemically modified and damaged bases during NER? Do they capture a base that is already flipped out, or do they drive the process of base-flipping?

It is evident from experiments that the time scale of base-flipping lies in the range of milliseconds to several hundred seconds or more. Hence, rare event methodology is required.

Some of the earliest investigations using molecular mechanics employed the FLEX force field in combination with the energy optimization program JUMNA (junction minimization of nucleic acids).^73−76^ Subsequent studies combined all-atom force fields such as AMBER (assisted model building with energy refinement) and CHARMM (chemistry at Harvard macromolecular mechanics) with umbrella sampling.^77^ This approach requires a predefined reaction coordinate (or order parameter), involving backbone torsions, distance and dihedral restraints.^43,67,78,79^ For biomolecular reactions that involve a complex rearrangement of atoms, order parameters may introduce bias,^80^ and regions of configuration space that are separated by large barriers can be incorrectly lumped together.^81−83^ Conformational changes orthogonal to the reaction coordinate can also be important.^84^

An alternative approach is to use collective variables. Conformational flooding^85^ and metadynamics^86^ simulations have exploited this methodology.^87^ An adaptive sampling algorithm, in which the simulation is guided back and forth to obtain multiple paths, has also been used to study base-flipping,^84^ while transition path sampling (TPS) provides another approach that requires order parameters.^88^ Finally, two other schemes have been employed that make it possible to explore the landscape orthogonal to the reaction coordinate, namely the on-the-path random walk method,^89^ which is a generalized ensemble sampling scheme, and selective integrated tempering sampling.^53^

The potential energy landscape (PEL) framework used in the present work does not employ reaction coordinates. In recent studies,^90−93^ we have successfully exploited the PEL framework to probe complex conformational transitions in nucleic acids, and rationalize key experimental findings. We note that the order parameters used for analysis during this investigation were only used in postprocessing to identify DNA duplex structures with a particular base present in an extrahelical state. However, the calculation of rates does depend on the definition of reactant and product, as discussed below.

## Methods

A DNA duplex with the sequence *d*(GA)_6_ was first constructed using the nucleic acid builder (NAB) program in AMBER18.^94−96^ Here, *d* stands for double stranded, where the complementary base pairing is implicit. The duplex was modeled using the symmetrized version^97^ of the AMBER99BSC0 force field^98−101^ along with torsional Olomouc corrections (χOL4).^102^ Symmetrization ensures that the permutational isomers have the same energy. The solvent and salt effects were treated implicitly using a generalized Born model (GB-OBC) based on the parametrization of Onufriev, Bashford, and Case.^103,104^

This sequence has been analyzed by Giudice et al., who studied base-flipping using umbrella sampling.^67^ We chose the same sequence to compare our results with the existing simulation study. Although there have been studies in the past describing the unusual structures adopted by oligopurine·oligopyrimidine sequences,^105^ in our work we have considered a short sequence of 12 base-pairs in the canonical Watson–Crick double helical structure. The structures with bases in a flipped-out state were obtained by employing group rotation moves implemented within the global optimization program GMIN.^106−108^ The group rotation moves were performed by defining two different kinds of pivot points. The first pivot point is based on atoms C5′ and O3′ in the respective base. The second pivot point is based on atoms forming the glycosidic bond, i.e., C1′ and N9 for purines and C1′ and N1 for pyrimidines. We perform group rotation moves by defining these two kinds of pivot points for each base that needs to be flipped. In the present study we have focused on four central bases, i.e., adenine, guanine, cytosine, and thymine, flipped out one at a time, to reduce edge effects for these relatively small systems.^109,110^ We note that experimental studies using NMR have shown that the length of the duplex and sequence can affect the barriers and flipping rates.^111^

The center-of-mass pseudodihedral angle, CPDb, described by Song et al., was used to diagnose which base in the DNA strand is flipped out, to what extent, and toward which groove (i.e., major or minor).^112^ For discrete path sampling (see Supporting Information) we require two end points. One end point was chosen as the lowest energy structure with all bases paired and the other was chosen with a base flipped out. Discrete path sampling employs pathway searches between successive pairs of end points in parallel. The structures with different bases flipped out (found using GMIN) were fed into PATHSAMPLE. Finally, several low energy states with the base flipped out to the maximum extent were considered for calculating barriers and rates. The flipped-out states reported in Table 4 are the subset with the lowest barriers and maximum rate constants for base-flipping.

We first need to obtain an initial connected pathway between the selected end point minima. The discrete path sampling approach was used to find pathways in terms of local minima and the transition states that connect them.^113−116^ The algorithms used within this procedure have been reviewed before,^82,117,118^ and we simply summarize the steps here:

- The doubly-nudged^119,120^ elastic band^121−124^ (DNEB) method was used to find candidate transition state geometries.
- Hybrid eigenvector-following was applied to obtain converged transition states from the candidates.^125^
- The limited-memory Broyden–Fletcher–Goldfarb–Shanno (L-BFGS)^126,127^ minimization algorithm was employed to obtain the two local minima directly connected by each transition state.^116^
- The missing connection algorithm^128^ was then used to choose pairs of minima for further double-ended connection attempts until a fully connected pathway between the initial and final states was obtained.

For a system of *N* atoms with 3*N* degrees of freedom, the potential energy landscape is a 3*N*-dimensional surface in a 3*N* + 1-dimensional space.^116^ To visualize this multidimensional surface we employ disconnectivity graphs^129^ where the potential (or free) energy is represented along the vertical axis. The equally spaced nodes located along this axis represent different superbasins. All the minima within the database are divided into these superbasins, which form disjoint sets. The barrier for interconversion of minima lying within the same superbasin is less than or equal to the threshold energy.^129,130^ Branches arising from these nodes represent the individual minima and terminate at the energy of a particular minimum.

Once an initial connected pathway has been found, large barriers may result due to incomplete sampling. Lower barrier pathways exist but have not yet been found, and further sampling is required. Various schemes^131^ to locate such pathways were employed in the present work. These schemes are implemented within the PATHSAMPLE program. Convergence of the sampling was monitored via inspection of disconnectivity graphs and computation of interconversion rates between target minima.^132,133^

Rates were extracted from the stationary point databases using graph transformation.^134−136^ The individual minimum-to-minimum rate constants were calculated using transition state theory in the harmonic approximation, as for local thermodynamic properties. The reactant state was considered to be the closed WC base-paired state, and the product state was taken to be an open state with one of the bases flipped out. The inverse rates in the backward and forward direction were taken as the lifetime of product and reactant, i.e., open and closed states, respectively.

## Results and Discussion

### Geometric Characterization of Base-Flipping

An order parameter was required to diagnose which base in the DNA strand was flipped out, to what extent it was flipped out, and toward which of the major or minor grooves it was oriented. The geometric parameters used for studying base-flipping are generally based on distances, angles and dihedrals, such as the distance between the N1 atom in purine and the N3 atom in pyrimidine.^137,138^ This parameter was also used in a recent computational study in which the authors compared their calculations with NMR observations without assuming that flipping was favored toward a particular groove.^84^ An alternative distance parameter is based on hydrogen-bonding atoms.^53^ The base plane rotation angle has also been employed.^76,79^

For these distance and angle parameters, it is not possible to identify whether the base has flipped toward the major or minor groove. While a larger distance indicates an open base, it is not possible to say exactly which of the two bases in a base-pair has flipped out, or if both the bases have flipped out simultaneously.^2^ To overcome this limitation, dihedral angles were used to classify flipped-out states.^67^ Several previous studies^43,112^ have utilized pseudotorsions, which successfully predict the flipping out process, and indicate in which direction the flipping occurs. Although there might be some issues when using these pseudotorsion angles as reaction coordinates, in our work we have only used them in postanalysis. Usually, one point in the dihedral is taken to be the base-pair flanking the base of interest, or the target base that is flipped out. This formulation emerges from one of the earlier studies on an adenine bulge.^139^ The first dihedral that was widely utilized was the center-of-mass (COM) pseudodihedral, also called the CPD.^43^ However, later it was found that this definition suffered from several limitations, i.e., conformations with similar CPDs differed significantly.^112^ Song et al. later defined another set of dihedrals: CPDa and CPDb. The CPDb dihedral was reported to work better and hence was used during the present analysis. The four sets of atoms considered for CPDb are the two base-pairs flanking the base of interest, the phosphate group on the 3′ side of the flipping base, the phosphate group on the 5′ side of the flipping base, and the ring atoms of the flipping base. It is important to note that in the case of purine flipping, only atoms of the five-membered ring are considered for the fourth point in the dihedral.^112^ Additionally, one of the possible reasons for CPDb being a better order parameter is that the second and third points in the CPDb dihedral lie close to the target base, whereas in CPDa the sugars of the neighboring base that are considered as the second and third points lie further away from the target base.

There are several torsion angles defined within the DNA backbone: α, β, γ, δ, ϵ, and ζ, and the glycosidic torsion angle χ.^140^ The changes in these dihedrals as a function of base-flipping have also been investigated in the past.^43−45,67,141^ However, these torsion angles have not been evaluated in the present study, primarily because the DNA structures that have been sampled in the current database may have local distortions in the backbone. Analyzing torsion angles localized on the flipping base, when any part of the entire backbone may be deformed, produces ambiguities. Analysis of the torsional profiles with base-flipping could provide important information about which torsions are crucial in this process. Furthermore, it could also yield information about any potential force field artifacts.

More recently, the solvent-accessible surface area (SASA) has been calculated to compare computational studies with NMR observations,^142,143^ highlighting limitations of NMR imino-H exchange results. Even when a base is flipped out slightly (approximately 30°), its SASA is large enough for the imino-H to be exchanged with the solvent.^2^ The magnitude of error in SASA calculations can be comparable to the SASA itself,^144−149^ which makes it less useful as an order parameter.

Energetic parameters are based upon the interactions between different atoms in the system, such as the flipping base and its partner, stacking interactions between adjacent bases, and interactions between hydrogen-bonding groups.^67,88^ The interaction between the base of interest and solvent has also been investigated using hydration number and solvent distribution.^67,88^ However, energetic factors were not employed in the present work.

The CPDb angles for the structures in the database were calculated using the AMBER trajectory analysis tool CPPTRAJ.^150^ The SASA of the N1 atom in guanine and N3 atom in thymine were calculated using the parameter optimized surfaces (POPS) program.^147−149^ These specific atoms were chosen because they correspond to the imino-H, where the exchange can be monitored during NMR studies.

**Figure 1:**
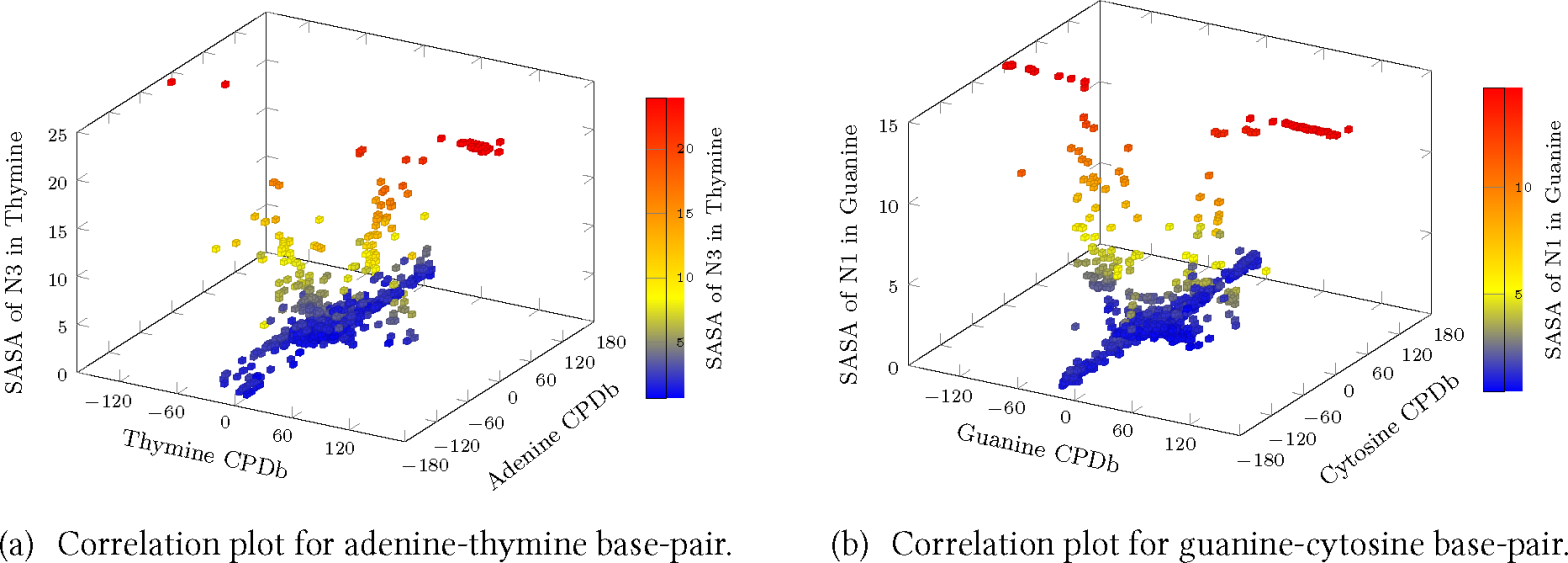
Correlation plots between the CPDb dihedral and SASA calculated using POPS. The CPDb dihedral angle is measured in degrees, and the SASA is measured in Å^2^.

The correlation between CPDb and SASA is shown in Figure 1. While similar correlation plots have been presented in the past,^151^ here, they are explicitly for CPDb. The key observation is that the SASA of N1 in G (or N3 in T) may be high even when CPDb is relatively small (around 30–40°). The SASA may also be high even when the partner base, i.e., C (or A) is flipped out. Therefore, SASA alone cannot be used to identify if a particular base is flipped out or if both bases are flipped out.

In the present work, positive CPDb angles correspond to the minor groove and negative CPDb angles to the major groove. These signs are the reverse of what has been reported before^112^ due to the definition of the CPDb dihedral and the method used to calculate dihedral angles implemented within the CPPTRAJ tool.^150^

### Topography of Energy Landscapes

**Figure 2:**
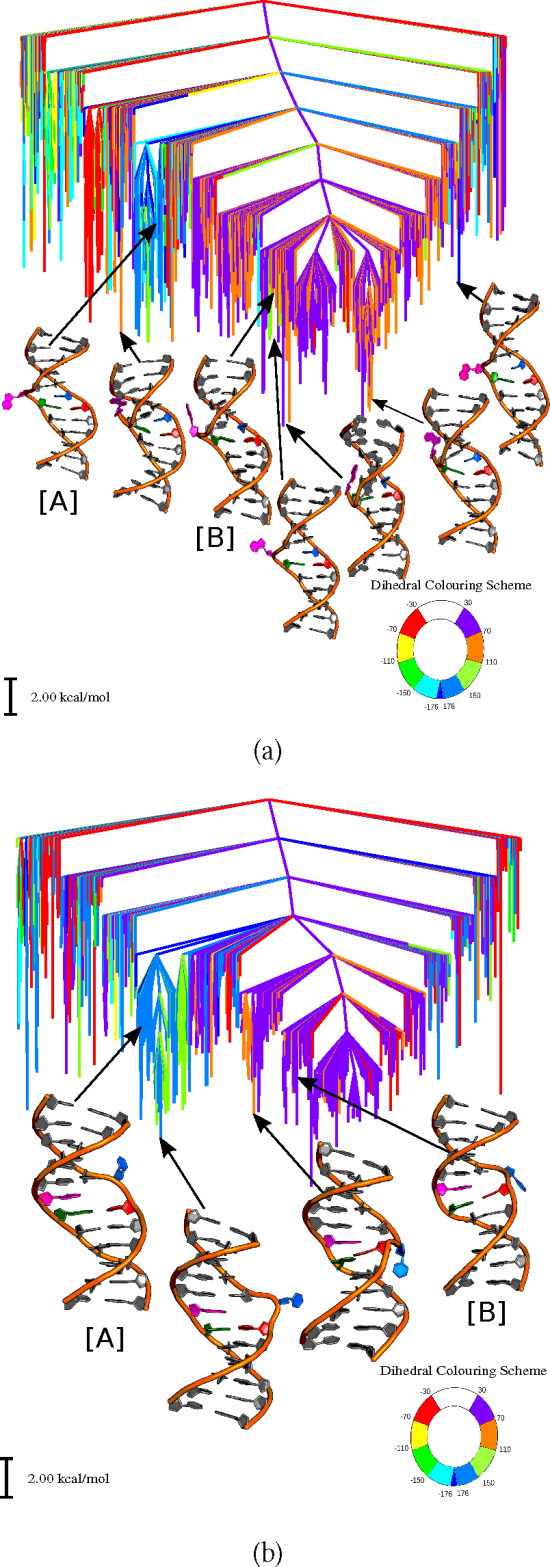
Free energy disconnectivity graphs for (a) adenine and (b) thymine bases flipped out of a DNA duplex at 300 K. The DNA structures labeled as [A] and [B] in the graphs represent the final flipped-out states considered in the plots in Figure 7. [A] and [B] represent the base flipped out via major and minor grooves, respectively.

**Figure 3:**
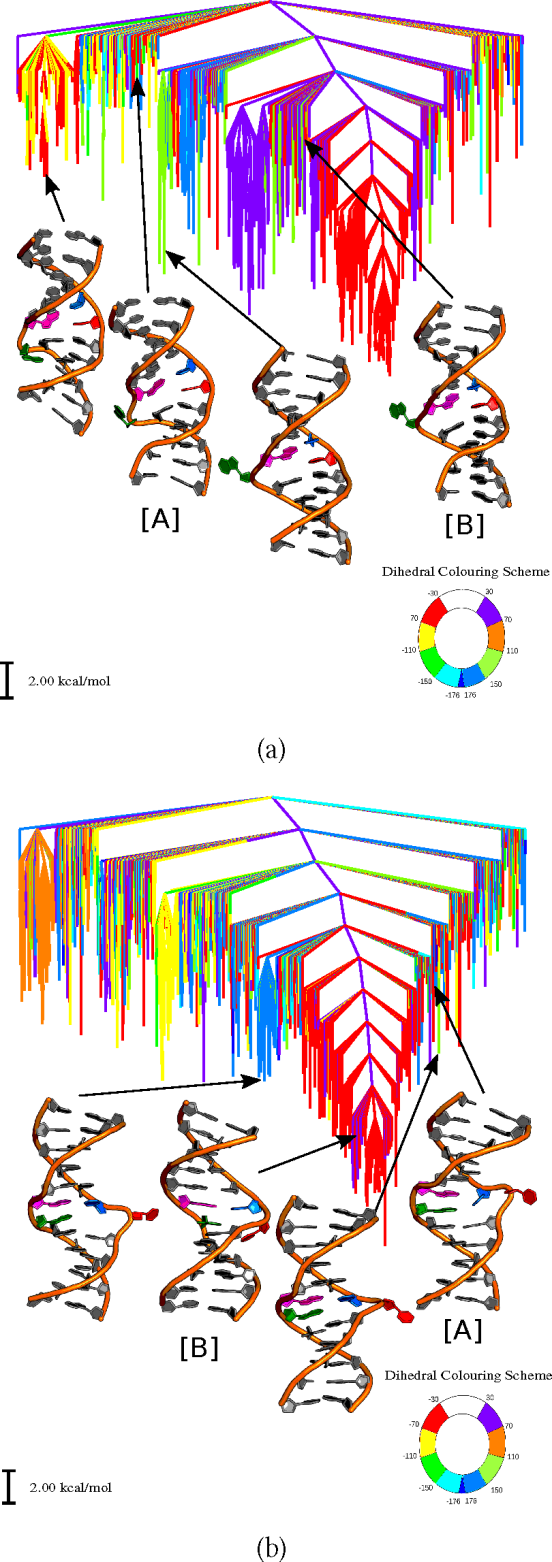
Free energy disconnectivity graphs for (a) guanine and (b) cytosine bases flipped out of a DNA duplex at 300 K. The DNA structures labeled as [A] and [B] in the graphs represent the final flipped-out states considered in the plots in Figure 7. [A] and [B] represent the base flipped out via major and minor grooves, respectively.

**Figure 4:**
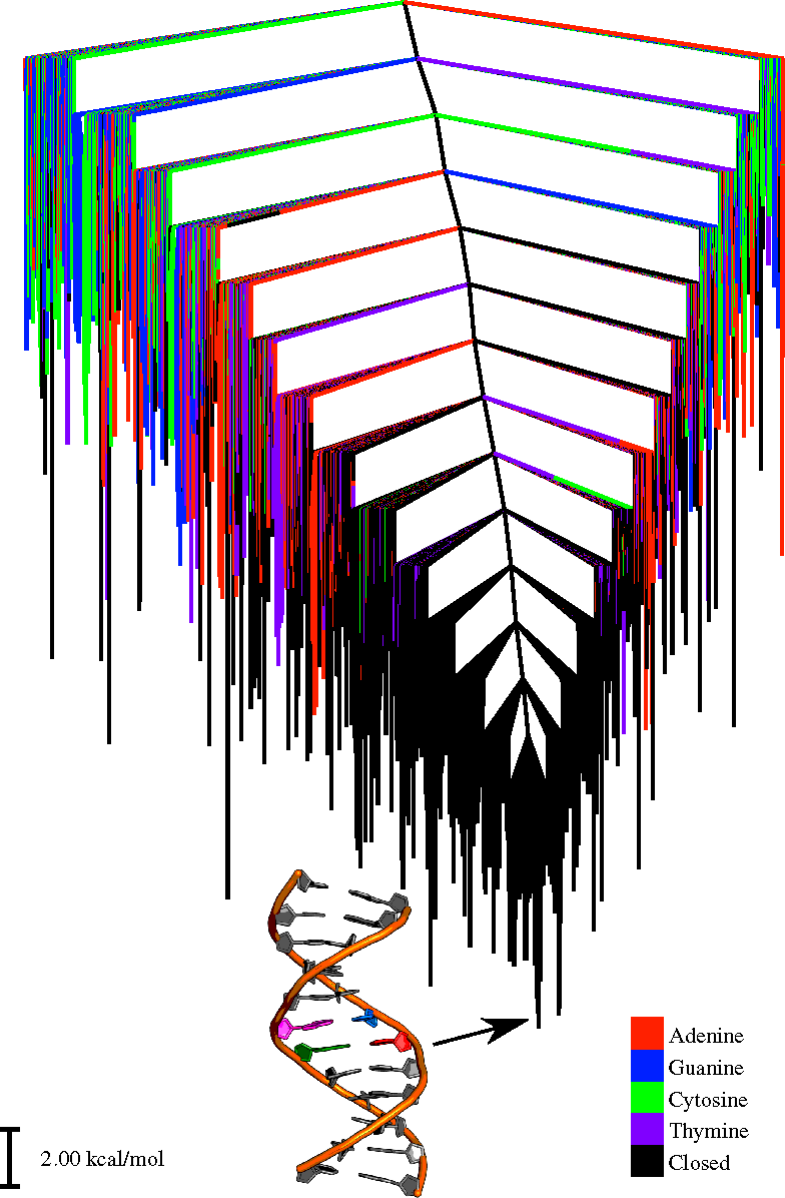
Free energy disconnectivity graph (at *T* = 300 K) including closed states and single base flips for all the four bases. The bases adenine, guanine, cytosine and thymine have been considered to be open if their CPDb dihedral is greater than 30 or less than −30 degree. The disconnectivity graphs in Figures 2 and 3 represent the landscape for flipping of individual bases separately. The same landscape is shown with different colors for minima featuring the four alternative flipped-out bases.

The free energy landscapes for flipping adenine, guanine, cytosine and thymine are shown using four disconnectivity graphs (Figures 2 and 3),^152,153^ colored on the basis of the CPDb dihedral angle for individual bases and an overall disconnectivity graph for the entire landscape (Figure 4). The graphs in Figures 2 and 3 were constructed by removing flipped-out minima for three of the four bases in each case.

Interestingly, the distribution of dihedral angles in the free energy landscapes of adenine and guanine is similar to that of their partner bases, i.e., thymine and cytosine. For both adenine and thymine, the bases flipped out slightly toward the minor groove lie at the bottom of the energy landscape. In contrast, for guanine and cytosine, the bases flipped out by small angles (30–60°) along the major groove lie lowest. However, for guanine, some of the minima with bases flipped into the minor groove are also low in energy. Similarly, for thymine, some of the configurations with bases flipped by small and large angles into the major groove are relatively favorable. Purines, i.e., adenine and guanine, apparently may prefer to flip via the minor groove. The local distortions of the backbone and widening of the minor groove facilitate this pathway for the sequence under consideration. We find that configurations with the bases flipped out by large angles via the major groove usually have lower free energy than for a minor groove pathway. This observation is consistent with previous work, which reported that the energy of bases flipped out via the minor groove was slightly higher than those that had flipped via the major groove. However, spontaneous conversion between the two flipped-out states was not possible, as the backbone conformations differed significantly.^79^

In all four disconnectivity graphs, minima with similar dihedral angles are found at different energy levels. There are various possible explanations for this observation. First, each color represents a range of angles spanning over 40°. This spread implies that the same colored branch present in a higher energy region may have the base flipped out by a larger angle. This possibility was checked by decreasing the range of angles grouped together and recoloring the disconnectivity graphs. Second, for the base flipped out toward the major groove, the pathway that was sampled in the database might involve the base flipping via the minor groove, and by traversing an angle greater than 180°, the base could have reached a dihedral associated with the major groove. A similar case was observed for bases flipped out toward the minor groove, where the pathway was found to be via the major groove. In both cases, the minima corresponding to such pathways lie higher in energy. In future work, it would be interesting to check if there is any difference in the backbone conformations of the bases flipped via the major and minor groove, as suggested in a previous study.^79^ Third, a base flipped out by a smaller angle may be located higher in energy. This result is possible when the pathway involves an intermediate state in which the base was flipped out by a larger angle, and then returned back to the final smaller angle. This effect may indicate a need for further sampling. Fourth, for a base flipped via the minor groove, the minima that are located toward the bottom of the graph usually have their backbones distorted, and a broad and distorted minor groove. In the absence of such distortions, the pathways lie higher in energy. In future work, we will examine the impact of bending on the energy landscapes for base-flipping more systematically.

It is evident that local distortions of the DNA backbone and subsequent broadening of the minor groove is necessary to facilitate base opening via the minor groove pathway. A similar trend has been reported in a previous study.^154^ Bending and opening of bases may be synergistically related, i.e. the bent backbone decreases the barrier, and the base-flipping makes the backbone more flexible, facilitating further bending.^154^ Bending decreases the energy needed to overcome the base–base interactions. In a bent backbone, there may be an accumulation of energy in the form of strain, which base-flipping may help to relieve.^73^ We note that the CPDb dihedral is no longer reliable in classifying whether the base lies toward major or minor groove side when the backbones are bent. In addition, for angles close to 170°, it is difficult to classify whether the base has followed a major or minor groove pathway. Again, this ambiguity is because of associated distortions observed at some stage during most of the base flipping pathways.

A recent study suggested that it is possible to have comparable barriers for adenine and thymine flipping in a DNA duplex.^84^ In the present work, the barrier for flipping adenine is higher than for thymine for major groove flipping pathways. However, the barriers for flipping A and T by large angles are comparable for minor groove flipping pathways. In an earlier report the barrier for flipping guanine was found to be higher than for cytosine.^43^ A contradictory observation was made in a subsequent study,^155^ and another recent computational investigation again suggests that cytosine is more prone to flipping in a GC base-pair.^84^ Results from the conformational flooding approach suggested that the barriers for flipping guanine and cytosine were comparable for the minor groove pathway. In the present work, the barrier for guanine flipping via the minor groove was found to be comparable to cytosine flipping via the major groove.^85^ The barrier for spontaneous flipping of undamaged bases was reported to be comparable by Zheng et al.^156^

To the best of our knowledge the only research in which exactly the same sequence was considered used umbrella sampling with a reaction coordinate that did not allow for DNA backbone distortions.^67^ Our estimated barriers may be quantitatively different from previous studies because the values depend on the sequence, the force field, the sampling method, and the choice of reactant and product states.^84^

### Mechanisms of Base-Flipping

**Figure 5:**
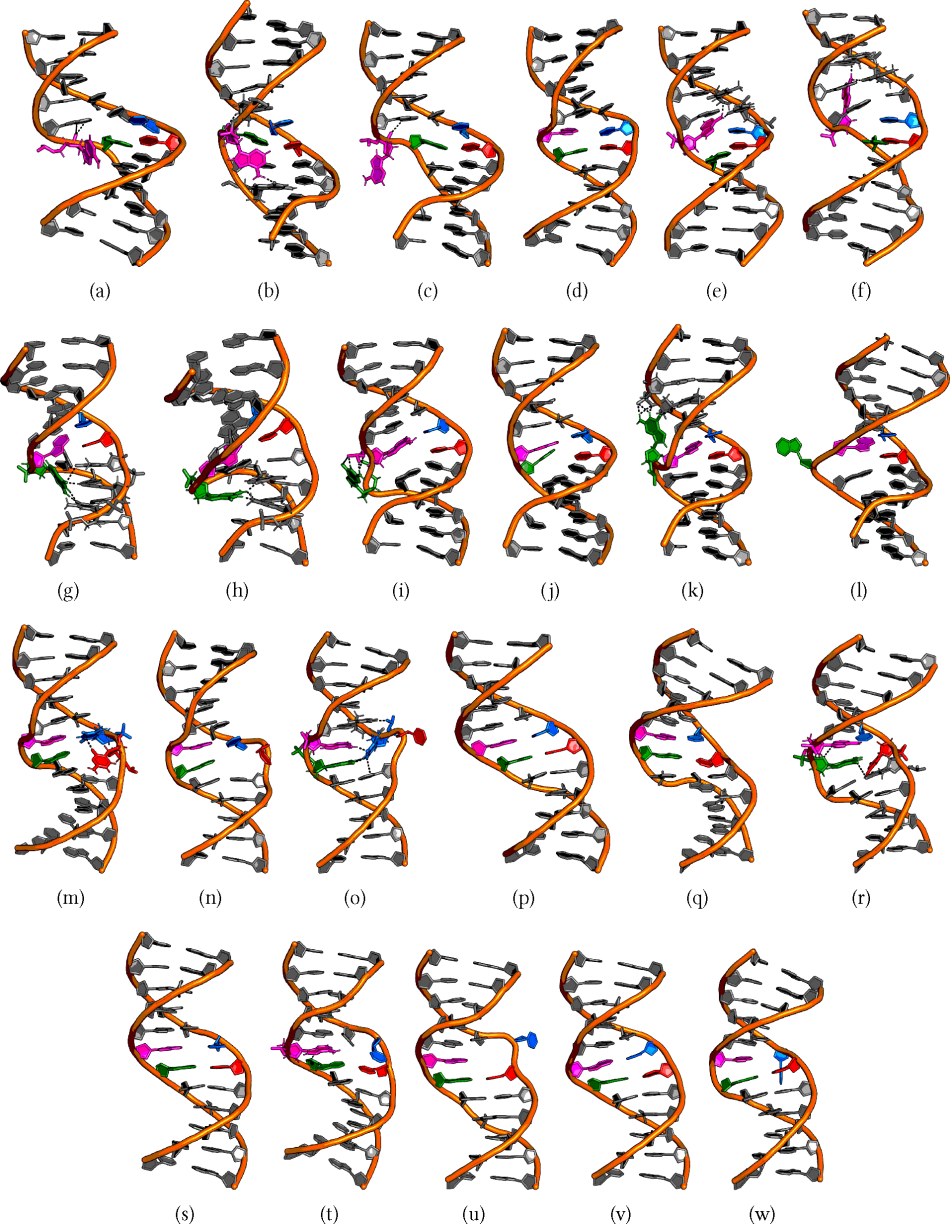
Snapshots of various steps in the base-flipping mechanism for individual bases. Adenine flipping via major (a–c) and minor groove (d–f), guanine flipping via major (g–i) and minor groove (j–l), cytosine flipping via major (m–o) and minor groove (p–r), and thymine flipping via major (s–u) and minor groove (v–w).

**Figure 6:**
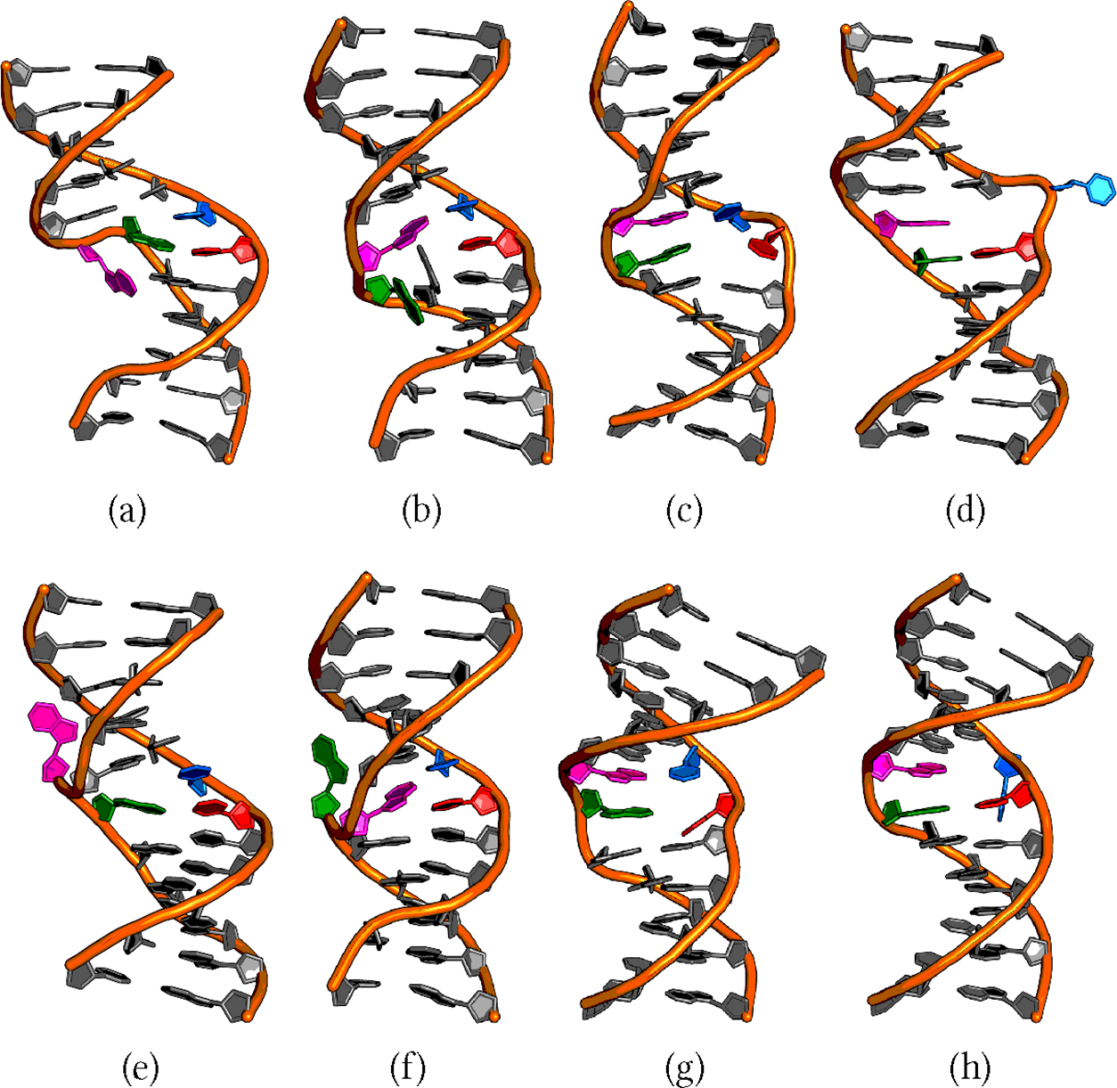
Panels a–d show adenine, guanine, cytosine and thymine flipping via the major groove, respectively, and panels e–h show adenine, guanine, cytosine and thymine flipping via the minor groove, respectively.

Base-flipping in a DNA duplex is a multistep process that involves a sequence of events occurring in a specific order. However, this order is not always strictly obeyed because several steps may occur in a concerted fashion. We distinguish eight distinct pathways for spontaneous flipping of adenine, guanine, cytosine, and thymine via the major and minor grooves, as summarized in Table 2 and Table 3. Table 2 lists the observed events, with an alphabetical code to define the pathways in Table 3. Some of the intermediates and transition states are also illustrated (Figures 5 and 6).

**Table 2:** Different Steps Observed During Base-Flipping with an Alphabetical Code Assigned to Each One, for Use in Table 3

event	description
	**breaking of hydrogen-bonds** may take place via,
S	linear separation of backbones, resulting in base plane elongation
Bb	bending of backbone containing the base being flipped out
C	concerted motion and bending (local distortion) of both backbones
U	unstacking of base with slight flipping
Dg	distortion of grooves: minor groove broadens and major groove narrows
D′g	the distorted groove reverts back to the original undistorted state
	**after the base first moves out** the following events may occur,
B	both backbones may bend further
B′	bent backbones straighten
N3	coupled motion of neighboring base on the 3′ side of the same strand, either to maintain stacking with base being flipped out, or to interact with the orphan WC partner
N5	coupled motion of base or base-pair on the 5′ side of the base being flipped out to maintain similar interactions as above
A5	the flipped base vertically aligns along its own backbone on 5′ side
A3	the flipped base vertically aligns along its own backbone on 3′ side
	**origin of sequence effects**
Ib′	the flipped-out base interacts with the backbone or/and bases of complementary strand that may be bent to further facilitate this interaction
Ib	the flipped base may interact with the backbone or/and bases of its own strand that maybe bent.
	**final flipping out**
F	the base may flip further out
R	the neighboring bases that moved during coupled motion may move back within the helix to maintain their own base pairing

**Table 3:** Observed Mechanisms for Flipping of Adenine, Guanine, Cytosine, and Thymine toward the Major and Minor Grooves^a^

		sequence of events								
base	groove									
adenine	major	S	C	Bb	U	A3	Ib	Dg	D′g	F
	minor	S	C	Dg	U	B	A5	Ib′	F	
guanine	major	C	Dg	U	N3	A3	Ib′	Ib	F	A5
	minor	C	Dg	U			A5	Ib	F	
cytosine	major	S	C	U	A5	N3	B		F	
	minor	C	Bb	Dg	N5	U	A5	Ib′	F	R
thymine	major	S	Bb	U	Ib	A3	A5	C	F	B′
	minor	S	Bb	Dg	U		A5	Ib	F	

a See Table 2 for the codes.

A key finding of the present analysis is that there may be a strong correlation between the sequence and the groove along which the base flips, so the flipping rate may be sequence dependent. When the base flips into the minor groove, it interacts with the base pairs on its 5′ side. In contrast, the flipping base usually interacts with the base pairs on its 3′ side when following the major groove pathway.

The events that occur during base-flipping can be classified into five broad categories: breaking of hydrogen-bonds, coupled motion of neighboring bases, alignment of the flipping base with its backbone, interaction of the open base with the backbone and other nearby bases, and bending (local distortion) of the backbones accompanied by distortion of grooves.

Recent computational studies suggest that base-flipping starts with the loss of hydrogen-bonding in the WC base-pair, i.e., base-pair opening.^84^ This opening may occur either by linear separation of strands, leading to base-plane elongation, or by twisting the base out of its plane.^88,89^ The separation of backbones by increasing the interphosphate distance before base pop-out has also been indicated in an earlier X-ray crystallography study on an enzyme–DNA complex.^157^ A similar observation has been made for cytosine flipping via the major groove. Conversely, strand separation during replication may be initiated by base-flipping.^43^ Once the base has opened, it may then unstack. Previous simulations have proposed that unstacking may occur after or simultaneously with the base-pair opening.^88^

The flipping of an individual base may be accompanied by the coupled motion of one or more of three other bases: the WC partner of the base, or the bases on its 5′ or 3′ sides. Earlier computational studies note that the WC partner base may follow the flipping base by moving toward the same groove. The possibility of the WC partner moving toward the opposite groove was also reported.^67,84^ Subsequent simulations have revealed that when purines flank the base that is being flipped out, they may move with it to retain the stacking interactions.^158^ A similar effect has been observed during the present analysis for guanine flipping via the major groove pathway. The adenine on its 3′ side breaks hydrogen-bonding interactions with its partner base and aligns its plane to stack with the flipping base. Several other studies have also shown the importance of stacking interactions in the case of purines.^67^ However, it remains to be seen whether the stacking interactions are essential when a purine is flipped out or when purines flank the flipping base.

Interestingly, the base on the 3′ side of the flipping base has been found to undergo significant distortion in both guanine and cytosine flipping via the major groove. This observation agrees with a previous study in which a similar distortion of bases on the 3′ side of the damaged base was reported.^159^ However, the base on the 5′ side has also been shown to undergo coupled motion when cytosine flips along the minor groove pathway. Moreover, when cytosine flips either via the major or minor groove, the partner base guanine changes its orientation so as to interact with the neighboring bases, such as thymine, on the opposite strand (Figures 5, parts o and r), either by forming hydrogen bonds, or by interacting with the sugar-phosphate backbone. In some cases when purine flips out, the partner pyrimidine base was found to change its orientation, either to better stack with the pyrimidine on its 5′ side (Figure 5c), or to come in close contact with the base on its 5′ side (Figure 5k).

Another remarkable experimental finding is the working of the enzyme thymine DNA glycosylase (TDG), which flips out T from a GT mismatch that has been formed from deamination of methylcytosine. How this enzyme knows the history of the nucleobase it interacts with is unknown.^42^ A recent F-NMR study has shown that there may be a link between the sequence that is more prone to methylation, and a similar sequence that has been observed to interact favorably with TDG.^42^ In any case, the enzyme differentiates between T in GT from the T in AT. This observation suggests an important role for the WC partner of the base being flipped out.

To the best of our knowledge, this is the first report linking major and minor groove pathways for base-flipping with the alignment of the base toward the 3′ or 5′ side of its backbone during an intermediate step, which shares some similarities with the e-motif structure first reported by Gao et al. and seen during recent computational work using the CHARMM27 force field.^87,160^ It consists of two cytosines in a CC mismatched base-pair aligned toward the 5′ side of their respective backbones, with simultaneous flipping toward the minor groove. While this process is beyond the scope of the current investigation, in which only one base is flipped out at a time, it is worth highlighting that when any of the four bases flips toward the minor groove, the base aligns itself along its own backbone on the 5′ side. In contrast, when the base flips via the major groove pathway, it usually aligns itself toward the 3′ side of its own backbone. However, for cytosine and thymine, both alignments have been observed when the base follows the major groove pathway.

An interesting hypothesis is that when the alteration in sequence on the 5′ side of the base being flipped out has a significant impact on the rates, the base is flipping via the minor groove pathway. Experimental confirmation could be achieved using a minor groove ligand and blocking the minor groove pathway. If the alteration in sequence on the 5′ side then has a limited impact on rates, that would be consistent with the hypothesis. Similarly, when the sequence on the 3′ side of the base is important, the base is probably following the major groove pathway. However, this hypothesis is subject to several caveats, highlighted below.

A computational study using the conformational flooding method suggested that, as the base cytosine flips out via the major groove, it first interacts with the backbone of the complementary strand and then with its own backbone.^85^ In particular, the amino group in the nucleobase interacts with the anionic phosphate groups in the backbone.^79^ The present work suggests similar interactions when purines are flipped out. Figure 6 depicts the polar contacts between the hydrogens of the amino group and the oxygen of either a nearby base or the sugar or phosphate group in the backbone.

One of the main causes of sequence effects is the interaction of the flipping base with the nearby bases.^43,67,161^ However, these nearby bases are not limited to adjacent bases or next-nearest neighbors, but include more remote bases.^43^ We observed polar contacts between the flipping base and its third- and fourth-nearest neighbors. This effect is evident in the following pathways: adenine flipping via the minor groove, and guanine flipping via the major and minor groove (Figure 5).

Although most computational studies report that flipping via the major groove is more favorable, several experiments have hypothesized that, since the enzymes interact with the DNA double helix from the major groove side, the base may prefer to flip toward the minor groove.^4,78,162,163^ Several reports have investigated base-flipping from a bent or unwound DNA duplex. A recent study using the CHARMM36 force field suggested that minor groove flipping may be favored by bent DNA.^154^ In fact, there is an early report on DNA untwisting and bending, indicating that, as the DNA bends toward the major groove, its minor groove widens, thereby facilitating base opening via the minor groove pathway.^73,76^

### Kinetics of Base-Flipping

Base-flipping rates have been determined using several experimental techniques. NMR imino-H exchange studies indicate that the lifetimes of AT and GC base-pairs are 1 to 5 ms and 10 to 50 ms, respectively.^37,38^ AFM investigations suggest that the AT base-pair lifetime is of the order of seconds, even in the absence of stacking interactions.^54^ Another approach involves the formation of a host–guest complex. The lifetime of purines has been reported to be around 1000 s using these complexation studies.^57−59^ More recently, a ddFCS study was performed on a mismatched base-pair, and the lifetime was found to be of the order of several seconds,^53^ differing from NMR results by 4 orders of magnitude. The fundamental question that arises from these observations is, what pathways and open states are sampled by different experimental techniques that lead to the difference in base-pair lifetimes?

The equilibrium constant for base-flipping has been determined in several computational studies.^2,43,79,164^ Umbrella sampling suggested that “NMR imino proton exchange experiments on duplex DNA primarily monitor the opening of purine bases”^155^ because the calculated rates for purines were significantly higher than for pyrimidines.^155^ In contrast, another umbrella sampling investigation, utilizing a polarizable force field, highlighted the importance of sequence effects and suggested that there are cases when the rates of flipping pyrimidines are higher than for purines.^165^

The present analysis estimates flipping rates of different bases by different extents along the major and minor grooves. Interestingly, the equilibrium constant of flipping guanine via the minor groove is significantly higher than via the major groove (Table 4). This difference may be attributed to the increased stabilization possible on the minor groove side, where the flipped base can interact well with neighboring base pairs. The rate constant for opening guanine via the minor groove is small, and the equilibrium constant for opening is high, because the corresponding flipped-out state is relatively stable, which decreases the rate of base closing. In a previous investigation on uracil DNA glycosylase (UDG), it was found that the enzyme increases the equilibrium constant for AT bp opening by stabilizing the open state.^40^ Another NMR study revealed a similar trend.^166^

**Table 4:** Kinetic Data for Base-Flipping^a^

Base	J or N	CPDb dihedral angle	barrier for opening	barrier for closing	rate of opening (s^–1^)	lifetime of closed state (s)	rate of closing (s^–1^)	lifetime of open state (s)	equilibrium constant
A	J	–172.11	25.51	10.60	1.09 × 10^–8^	9.17 × 10^7^	6.37 × 10^1^	1.57 × 10^–2^	1.70 × 10^–10^
	N	126.56	15.35	5.04	1.62 × 10^–2^	6.17 × 10^1^	4.54 × 10^4^	2.20 × 10^–5^	3.57 × 10^–7^
G	J	–47.99	15.50	1.47	1.49 × 10^–2^	6.71 × 10^1^	4.55 × 10^4^	2.20 × 10^–5^	3.27 × 10^–7^
		–122.02	29.19	3.07	1.26 × 10^–12^	7.94 × 10^11^	1.62 × 10^–1^	6.18	7.78 × 10^–12^
	N	67.00	22.67	5.43	7.67 × 10^–8^	1.30 × 10^7^	8.96 × 10^–4^	1.12 × 10^–3^	8.56 × 10^–5^
		132.08	25.99	4.28	4.33 × 10^–10^	2.31 × 10^9^	8.96 × 10^–4^	1.12 × 10^–3^	4.83 × 10^–7^
C	J	–45.41	14.39	7.69	7.66 × 10^–2^	1.31 × 10^1^	4.97 × 10^3^	2.01 × 10^–4^	1.54 × 10^–5^
		–124.29	22.84	2.46	5.28 × 10^–8^	1.89 × 10^7^	1.02 × 10^4^	9.82 × 10^–5^	5.19 × 10^–12^
		172.92	26.07	5.52	2.37 × 10^–10^	4.22 × 10^9^	2.84 × 10^2^	3.52 × 10^–3^	8.34 × 10^–13^
	N	47.52	15.08	5.75	3.95 × 10^–2^	2.53 × 10^1^	4.55 × 10^4^	2.20 × 10^–5^	8.68 × 10^–7^
T	J	–46.83	11.19	0.80	1.63 × 10^1^	6.12 × 10^–2^	4.55 × 10^4^	2.19 × 10^–5^	3.59 × 10^–4^
		177.71	19.29	6.05	3.16 × 10^–5^	3.16 × 10^4^	4.82 × 10^3^	2.07 × 10^–4^	6.55 × 10^–9^
	N	76.30	14.39	7.70	7.66 × 10^–2^	1.31 × 10^1^	4.97 × 10^3^	2.01 × 10^–4^	1.54 × 10^–5^
		121.28	14.79	3.22	2.61 × 10^–2^	3.83 × 10^1^	4.97 × 10^3^	2.01 × 10^–4^	5.26 × 10^–6^

a The letters “J” and “N” in the second column stand for the pathway via major and minor grooves, respectively. The barriers for opening and closing are the free energy barriers at 300 K in kcal/mol.

It has been suggested that NMR studies analyze the rate of base-pair wobbling instead of full base-pair opening.^2,43,44^ In our calculations, the rates of base opening, and hence the lifetimes of bases in the closed state, for flipping thymine by small and large angles via the major groove differ by 6 orders of magnitude, whereas for thymine flipping by small angles into the minor groove, and large angles into the major groove, the rates differ by 3 orders of magnitude. Since this difference is close to the difference in rates observed using NMR and ddFCS studies, it is possible that NMR imino-H exchange studies monitor base-flipping through small angles, while ddFCS reports on larger angles.^53^

**Figure 7:**
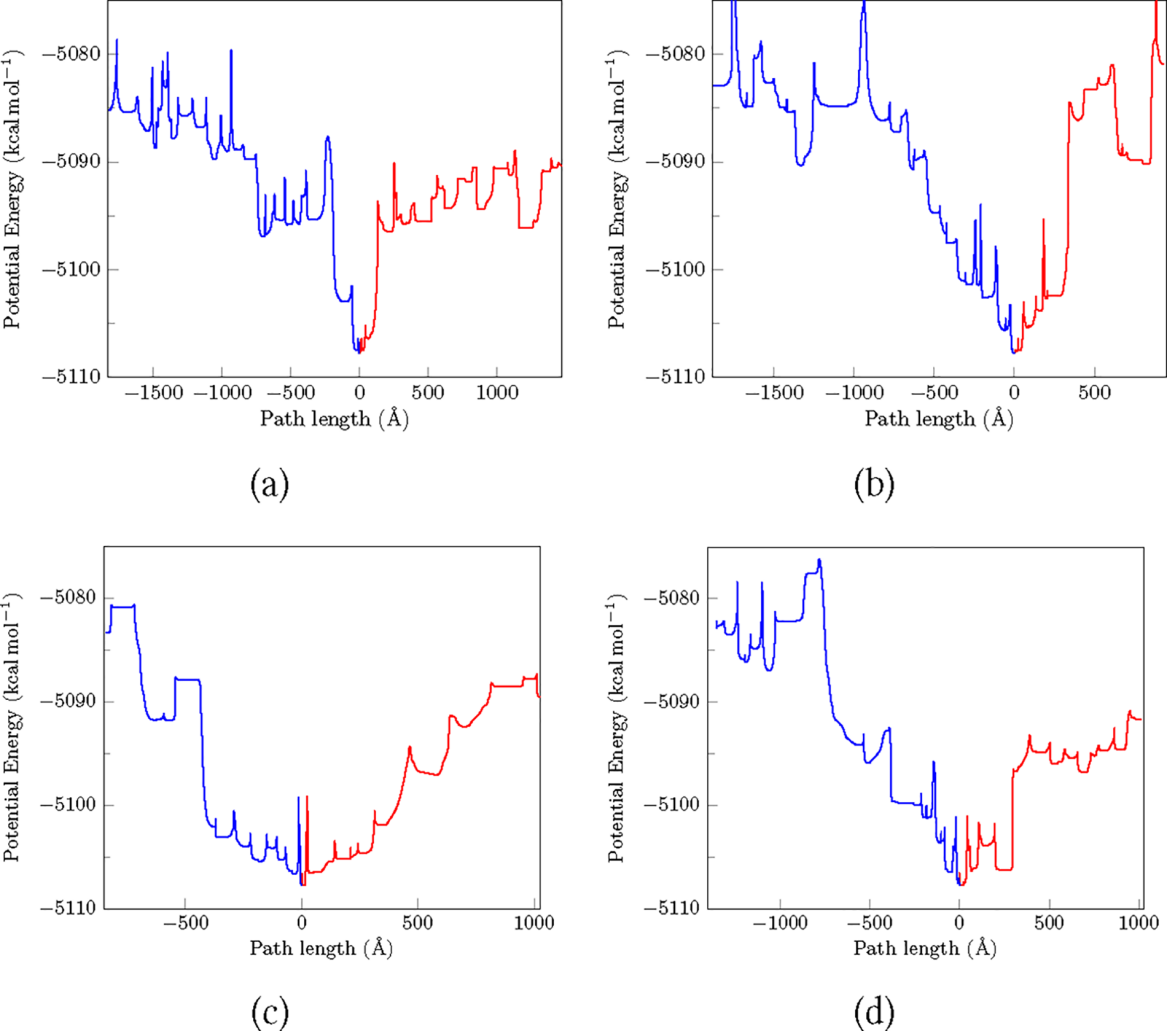
Potential energy as a function of integrated path length for flipping pathways of (a) adenine, (b) guanine, (c) cytosine, and (d) thymine along the major and minor groove. Positive and negative path lengths correspond to flipping along the minor and major groove, respectively. The pathways are between closed state (as shown within Figure 4) and open states labeled as [A] and [B] (Figures 2 and 3) for the base flipped out via major and minor grooves, respectively.

The potential energy change as the base flips out via major and minor grooves is plotted against the integrated path length in Figure 7. Different open states have been sampled along the two grooves. For cytosine, the open state considered for the minor groove has a smaller angle than the open state considered for the major groove.

Giudice et al. have previously reported free energy plots for the base-opening angle. They attributed the initial quadratic increase in energy to the breaking of hydrogen-bonds.^67^ The subsequent linear increase in energy was interpreted in terms of loss of stacking interactions as the base flipped out further. They also reported that purines preferred to flip via the major groove. The effective water bridging that was possible on the major groove side favored base-pair opening.^67^

In contrast, the present study suggests that the purines may prefer the minor groove pathway. This difference might be explained in terms of DNA distortions, which is permitted in the current work, but not in the previous study. Giudice et al. also discussed symmetric base-pair opening for pyrimidines, and found that it was then equally probable for the base to flip out via major or minor grooves.^67^ Indeed, this is the case for thymine in our analysis. However, most pathways that were sampled here involve base-flipping via the major groove for cytosine. Even for some dihedral angles corresponding to the base opened toward the minor groove, the pathway corresponds to the base moving by more than 180° via the major groove.

### Limitations of the Present Study

The present study comes with its own set of limitations. Parametrizing a force field to represent all possible conformations of a large macromolecule like DNA is clearly challenging,^167^ and different barriers for flipping the same base in the same sequence have been reported using alternative force fields.^110,168,169^ When the base flips out, its environment changes from hydrophobic within the helix to polyanionic, when it moves close to the backbone. Finally, it ends up in an aqueous environment.^165^ During this process, there may be a considerable change in the charge distribution of the nucleobase, which might be better represented by a polarizable force field.^165^

A recent X-ray crystallography study revealed the existence of a spine of hydration in the minor groove of DNA,^170^ and a computational study revealed that the water channel fills the gap left in the helix at the abasic site.^171^ In the current investigation, water has been modeled using an implicit solvent model. Comparison with explicit solvent will be considered in future work.

Biomolecules interact with water molecules as well as surrounding ions, and the salt concentration influences the free energies.^172,173^ In an experimental study, magnesium ions were added to an extract of human cells to facilitate DNA repair.^174^ Additionally, several investigations have provided evidence for the influence of ions on the width of the minor groove.^172,173^ A recent study has also confirmed that the barrier obtained for base-flipping varies with the salt concentration.^169^

The same base in alternative sequences can behave differently.^44,175^ A prominent example of this effect is provided by AT-tracts, which form when four or more AT base-pairs occur together without a 5′-TA-3′ step in between. The lifetime of an AT base-pair in AT-tracts is much higher than for an isolated AT base-pair. Conversely, the lifetime of a GC bp in GC-tracts is significantly lower than for an isolated GC bp. Hence, the opening rates of bases in AT-tracts are slower than in GC-tracts.^68,69,109,111,176^

The sequence also influences the dimensions of grooves in a DNA helix.^177,178^ In particular, sequences rich in AT base-pairs have a relatively narrow minor groove,^179−181^ and NMR studies indicate that the methyl group on the fifth carbon of thymine is the underlying cause.^182^ The sequence also determines the stacking of bases within the duplex^183^ and the water-mediated hydrogen-bonding interactions.^43^ In general, the pathways, barriers, and rates for base-flipping all depend on the sequence.^184,185^ The mechanisms presented in the present work are expected to be generic, however, the details may be specific for the sequence under consideration. In particular, we expect that base opening toward the major or minor groove is likely to be sequence dependent.

## Conclusions

Perhaps the most interesting hypothesis presented here is the relationship between the sequence and the direction of base opening, i.e., toward the major or minor groove. In particular, base-flipping along the minor groove pathway was found to align toward the 5′ side of the backbone. The base was found to align toward the 3′ side of the backbone when flipping along the major groove pathway. However, in some cases for cytosine and thymine, the base-flipping along the major groove pathway was also found to align toward the 5′ side. The sequence effects may be caused by interactions of the flipping base with the neighboring base-pairs on the side toward which it aligns.

Another observation is that purines might prefer to flip via the minor groove pathway. While this suggestion contradicts previous studies,^67^ in which purine flipping toward the minor groove was found to be restricted due to steric reasons, we found that bending and subsequent distortion of the minor groove lower the barrier and promote base-flipping toward the minor groove. A special case occurs when guanine flips along the minor groove pathway because the flipped-out state is relatively stable. The equilibrium constant for base-opening is high and correlated with the reduced rate of base-closing.

Our results may be compared with two experimental observations. First, in an earlier study on the CC mismatched base-pair, both the Cs were found to be aligned toward the 5′ direction when the bases were opened toward the minor groove.^87,160^ We find that the alignment toward 5′ is associated with flipping toward the minor groove for all four bases: A, G, C, and T. Second, NMR studies show that AT-tracts have a low rate of base-pair opening,^68^ and crystal structure studies reveal that AT-tracts have a narrow minor groove.^180,181^ A computational study reveals that NMR imino proton exchange monitors flipping of purine bases.^155^ These findings may be connected by the preference of flipping via the minor groove pathway for purines, including adenine.

In future work, we will investigate the influence of stretching, bending, and twisting on the energy landscape for base-flipping. The correlation between sequence effects and the direction of opening is another important question that needs to be resolved. One approach would be to study various sequences that are already known to interact with specific enzymes experimentally.

## Supporting Information Available

Detailed explanation of the discrete path sampling approach, calculation of free energy and rate constants for multistep pathways, input files for use with the PATHSAMPLE program and OPTIM interfaced with AMBER12, and the CPPTRAJ script used to calculate the CPDb dihedral, along with a figure defining the CPDb dihedral; free energy disconnectivity graphs at 300 K to distinguish flipping via major and minor groove for adenine, guanine, cytosine, and thymine flipped out one at a time (PDF)
