# Supplementary material for: Energy Landscapes for Base-Flipping in a Model DNA Duplex
Source: J Phys Chem B. 2022 Apr 15;126(16):3012–28. doi: 10.1021/acs.jpcb.2c00340 (PMC9098180; doi:10.1021/acs.jpcb.2c00340)
Supplement: Supplementary file 1 — jp2c00340_si_001.pdf [file jp2c00340_si_001.pdf]

Energy Landscapes for Base-Flipping in a Model DNA Duplex

Nicy,[*,†] Debayan Chakraborty,[*,‡] and David J. Wales[*,†]

†*Yusuf Hamied Department of Chemistry, University of Cambridge, Lensfield Road, Cambridge, CB2 1EW, U.K.*

‡*Department of Chemistry, The University of Texas at Austin, Austin, Texas 78712, United States*

E-mail: nn320@cam.ac.uk; debayan.chakraborty@utexas.edu; dw34@cam.ac.uk

Supporting Information

Discrete path sampling

The potential energy landscape (PEL) framework involves exploring a chemical reaction on a high dimensional potential energy surface (PES) using the discrete path sampling (DPS) technique. The landscape is first coarse-grained in terms of stationary points: local minima that specify the stable structures in which the system may exist, and first-order saddle points that correspond to transition states.[1,2] A discrete path between two endpoints consists of a series of minima connected by intervening transition states. For each stationary point, the normal mode frequencies are obtained from the eigenvalues of the mass-weighted Hessian matrix. A local minimum has non-negative normal mode frequencies, whereas a transition state has a single imaginary frequency.[3]

The OPTIM code[4] interfaced with the AMBER12 package was used for all geometry optimisations, transition state searches, and normal-mode analysis. Local minimisations were performed using the Limited-Memory Broyden Fletcher Goldfarb Shanno algorithm (LBFGS).[5] Candidate

transition states between a pair of minima were obtained using the doubly-nudged elastic band (DNEB) method.[6–8] Transition state candidates were further refined using hybrid eigenvector-following.[9,10] After each cycle of connection-making attempts, a large number of intervening minima and transition states may be found. Next, the missing connection algorithm is employed to construct a priority list of of connection attempts based on an appropriate edge-weight metric.[11,12]

After an initial discrete path was found between two endpoints, the stationary point database was further expanded using the PATHSAMPLE[13] program. In the current work further sampling was conducted using the CONNECTPAIRS and UNTRAP[14] schemes implemented within PATHSAMPLE. CONNECTPAIRS specifies pairs of minima for connection attempts. The main advantage of using the other keyword, UNTRAP, is that by specifying the minima at funnel bottoms faster energy pathways can be efficiently found for all the minima in the funnel. Consequently, an entire funnel lying higher up in the disconnectivity graph may be connected to lower energy. In contrast, CONNECTPAIRS requires individual minima to be specified, and connections are attempted only for those minima. However, CONNECTPAIRS was found to be much easier to use during the current work and was extensively employed for further sampling. UNTRAP was also used to connect one of the funnels lying higher up in the disconnectivity graph of adenine.

Once the disconnectivity graphs stopped changing appreciably with additional sampling, and consistent rates (i.e., showing negligible variation with additional sampling) were obtained for base flipping, sampling was assumed to be complete, and the landscape was deemed to be converged.[15,16]

An implicit solvent model was used to represent water. An explicit solvent model would have increased the degrees of freedom, making geometry optimisation computationally expensive. Explicit solvent would have slowed down sampling, since the database would include stationary points differing only in the conformation of water molecules. Various studies in the past, both for RNA and DNA, have demonstrated the usefulness of implicit solvent and how the results obtained from such simulations can be in close agreement with the more expensive explicit solvent simulations, and with experiments.[17–25]

Calculation of free energies

Statistical mechanics is used for calculating macroscopic properties from a microscopic point of view.[26] For example, the free energy $F_i(T)$ associated with a local minimum i at temperature T can be expressed as,[26]

$$F_i(T) = -k_B T \ln Z_i(T) \tag{1}$$

where k_B is the Boltzmann constant and $Z_i(T)$ is the partition function of minimum i at temperature T. This partition function can be calculated from the Laplace transform of the microcanonical density of states $\Omega_i(E)$, i.e.,[2]

$$Z_i(T) = \int \Omega_i(E) \exp^{-\beta E} dE, \tag{2}$$

where $\beta = 1/k_B T$. Further, a harmonic approximation to $\Omega_i(E)$ can be calculated using normal mode vibrational frequencies,[26]

$$\Omega_i(E) = \frac{n_i (E - V_i)^{\kappa-1}}{\Gamma(\kappa) \prod_{\alpha=1}^{\kappa} h\nu_\alpha(i)}, \tag{3}$$

where $n_i = 2N_\mathrm{A} N_\mathrm{B} \ldots / o_i$. Here, N_A is the number of atoms of type A in the system, A, B, ... are all the different kinds of atoms present in the system, o_i is the order of point group, κ is the number of degrees of freedom, $\Gamma(\kappa) = (\kappa - 1)!$, $\nu_\alpha(i) = \omega_\alpha(i)/2\pi$, and $\omega_\alpha(i)$ is the normal mode vibrational frequency.[26–28] Using equation 3 in 2, $Z_i(T)$ can be written as

$$Z_i(T) = \frac{n_i \exp^{-\beta V_i}}{(\beta h \bar{\nu}_i)^\kappa}, \tag{4}$$

where $\bar{\nu}_i = \left[\prod_{\alpha=1}^{\kappa} h\nu_\alpha(i)\right]^{1/\kappa}$ is the geometric mean of the vibrational frequencies associated with minimum i. These vibrational frequencies are obtained from eigenvalues of the mass weighted hessian matrix $\mathbf{h}$.[2,27–31] For a transition state, $\bar{\nu}_i$ is calculated by excluding the imaginary frequency associated with the negative eigenvalue of $\mathbf{h}$.[2]

For a system containing a constant number of particles N at constant temperature T and constant volume V, the canonical partition function $Z(T)$ was formulated using the Harmonic Superposition Approximation (HSA). The partition function includes contributions from basins of attraction of all the local minima i in the database, i.e.,[32]

$$Z(T) = \sum_i Z_i(T) . \tag{5}$$

Kinetic properties

The kinetics of a chemical reaction are described using rate constants. The rate constants can be calculated using Transition State Theory (TST), i.e.,[33–37]

$$k_i^\dagger(T) = \frac{k_B T}{h} \frac{Z^\dagger(T)}{Z_i(T)} \exp^{-\Delta V / k_B T} .$$

Here, $k_i^\dagger(T)$ represents the unimolecular canonical reaction rate constant from minimum i to transition state † at temperature T, k_B is the Botlzmann constant, h is Planck's constant, $Z^\dagger(T)$ is the partition function of the transition state, $Z_i(T)$ is the partition function of minimum i, and ΔV is the change in potential energy between the minimum and the transition state.

However, biomolecular reactions usually involve multiple minima-transition state-minima steps. In this case, the system is evolved using a linear master equation[38,39] which assumes that the overall dynamics are Markovian.[2] Markovian dynamics means that the rate of transition from one minimum to the next is independent of the pathway that was taken to reach the initial minimum.[2] The assumption behind this independence is that the reactant has enough time to reach a thermal equilibrium when it arrives at a particular state. Using the linear master equation, the change in occupation probability $p_a(t)$ of state a with time t, can be expressed as

$$\frac{dp_a(t)}{dt} = \sum_{b \neq a} \left[k_{ab} p_b(t) - k_{ba} p_a(t) \right] , \tag{6}$$

where k_{ab} represents the rate constant of the single step reaction from minimum b to a and k_{ba} represents the rate constant from minimum a to b where a and b are geometrically distinct.

Since, the pathway is multi-step and has intervening minima, a steady-state approximation may be used for intermediate minima.[3,40,41] However, biomolecular reactions are rarely single step with the reactant defined by a single minimum. It is usually necessary to define reactant and product using an ensemble of states, i.e., a set of minima. The master equation can now be rewritten for an ensemble of states A as,

$$\frac{dp_A(t)}{dt} = k_{AB}p_B(t) - k_{BA}p_A(t) \tag{7}$$

where p_A and p_B are the occupation probabilities of A and B, respectively.

$$p_A(t) = \sum_{a\in A} p_a(t) \text{ and } p_B(t) = \sum_{b\in B} p_b(t) \tag{8}$$

and

$$k_{AB} = \frac{1}{p_B^{eq}} \sum_{a\in A}\sum_{b\in B} k_{ab}p_b^{eq} \text{ and } k_{BA} = \frac{1}{p_A^{eq}} \sum_{a\in A}\sum_{b\in B} k_{ba}p_a^{eq}. \tag{9}$$

The above equation assumes a local equilibrium condition, i.e.,

$$p_a(t) = \frac{p_a^{eq}p_A(t)}{p_A^{eq}} \text{ and } p_b(t) = \frac{p_b^{eq}p_B(t)}{p_B^{eq}}. \tag{10}$$

The phenomenological rate constants k_{AB} and k_{BA} include sums over all the elementary transitions from $a \leftarrow b$, and $b \leftarrow a$ that lie on the boundary of regions defined by set A and B.[3]

To treat a multiple step chemical reaction, the steady-state approximation may be applied for intervening states i. In this case, the evolution of occupation probability for intermediate state i is assumed to be,

$$\frac{dp_i(t)}{dt} = \sum_a \left[k_{ia}p_a(t) - k_{ai}p_i(t)\right] = 0 \tag{11}$$

$$\Rightarrow p_i(t) = \frac{\sum_a k_{ia}p_a}{\sum_i k_{ai}}. \tag{12}$$

The steady-state rate constant from B to A (k_{AB}^{SS}) can now be written as,

$$k_{AB}^{SS} = \frac{1}{p_B^{eq}} \sum_{a \leftarrow b} \frac{k_{ai_1} k_{i_1 i_2} \ldots k_{i_n b} p_b^{eq}}{\sum_{j_1} k_{j_1 i_1} \sum_{j_2} k_{j_2 i_2} \ldots \sum_{j_n} k_{j_n i_n}}. \tag{13}$$

Equation (13) can be further simplified using the definitions of transition (or branching) probability p_{ab} as given in Equation (14), waiting time or inverse of escape rate or lifetime τ defined in Equation (15), and then committor probability defined in Equation (16).[2]

$$p_{ab} = \frac{k_{ab}}{\sum_{b'} k_{b'b}} \tag{14}$$

$$\tau_b^{-1} = \sum_{b'} k_{b'b} \tag{15}$$

$$C_b^A = \sum_{a \notin B} p_{ab} C_a^A \tag{16}$$

The committor probability from a minimum b to A is defined as the probability that a random walk starting from b will end up in a minimum contained in set A before visiting a minimum in set B.[41] Then, Equation (13) can be rewritten as,

$$k_{AB}^{SS} = \frac{1}{p_B^{eq}} \sum_{a \leftarrow b} p_{ai_1} p_{i_1 i_2} \ldots p_{i_n b} p_b^{eq} \tau_b^{-1} = \frac{1}{p_B^{eq}} \sum_{b \in B} \frac{C_b^A p_b^{eq}}{\tau_b}. \tag{17}$$

Here, B and A are the set of reactant and product minima and b and a are specific representative local minimum belonging to B and A, respectively.

When the steady-state approximation is not taken into account, i.e., the waiting time in intermediate minima is not negligible, the effective waiting time in the reactant state b increases and is given by t_b where $t_b \geq \tau_b$.[2] The non-steady state rate constant is defined as,

$$k_{AB}^{NSS} = \frac{1}{p_B^{eq}} \sum_{b \in B} \frac{C_b^A p_b^{eq}}{t_b}. \tag{18}$$

For multiple multi-step pathways between the reactant minima and product minima the rates are

calculated by making a graph out of the stationary point database. In the graph, each minimum is represented by a node and the minima that are directly connected by a transition state are represented using edges between the respective nodes of the minima.[42] The pathway that contributes the most to the rate constant is calculated using Dijkstra's shortest path algorithm.[11] The overall rate constant can be calculated efficiently using Graph Transformation,[41,43] which involves removal of intermediate minima with subsequent renormalisation of branching probabilities and waiting times of minima so as to conserve the mean first passage time, i.e.,

$$k_{AB}^{NSS} = \frac{1}{p_B^{eq}} \sum_{b \in B} \frac{p'_{Ab} p_b^{eq}}{\tau'_b}, \tag{19}$$

where p'_{Ab} is the renormalised branching probability from b to A and τ'_b corresponds to the renormalised waiting time for state b.

The linear master equation is solved by combining a matrix formulation with graph transformation, which proves to be even more efficient.[44]

Input files and keywords

A sample pathdata input file for extracting fastest pathway between two minima using the PATHSAMPLE program has the following keywords

TEMPERATURE 0.596

PLANCK 9.536D-14

EXEC A12OPTIM

NATOMS 760

COPYFILES perm.allow min.in coords.prmtop coords.inpcrd

COPYOPTIM

CYCLES 0

DIRECTION AB

DIJKSTRA 0

CONNECTIONS 1

ITOL 1.0D0

GEOMDIFFTOL 0.1D0

EDIFFTOL 1.0D-4

PERMDIST

AMBER12

The input file odata.connect for use with OPTIM contains the following keywords

UPDATES 1000

NEWCONNECT 100 3 20.0 40.0 80 2.0 0.001

NEWNEB 100 1000 0.1

NEBK 10

NOCISTRANS 160.0 DNA

DIJKSTRA EXP

DUMPALLPATHS

REOPTIMISEENDPOINTS

EDIFFTOL 1.0D-4

MAXERISE 1.0D-4 1.0D-2

GEOMDIFFTOL 0.1D0

BFGSTS 1000 20 200 0.01 100

BFGSMIN 1.0D-6

NOHESS

LPERMDIST 11 0.5 5.0 0.06

MAXSTEP 0.2

TRAD 0.5

MAXMAX 1.0

BFGSCONV 1.0D-6

PUSHOPT 0.2 0.001 100

STEPS 1000

BFGSSTEPS 60000

MAXBFGS 0.2

AMBER12 start

An explanation of the keywords contained in the pathdata and odata.connect files can be found at the PATHSAMPLE[13] and OPTIM[4] websites. Some of these keywords in odata.connect are explained here in the sequence in which they are used to generate a discrete path between two endpoints.

1. For checking the initial and final states

    - REOPTIMISEENDPOINTS checks the RMS (root mean square) force of the endpoint geometries and reconverges these endpoints if the RMS is above the threshold.
    - NOCISTRANS performs chirality and cis-trans checks on the two minima.
    - LPERMDIST is used for local permutational alignment of the minima.[45]

2. For connecting endpoints with images and springs

    - NEBK is used to specify the spring constant of the springs joining the images.
    - The arguments of NEWNEB instruct the OPTIM program about the maximum number of images, the maximum number of iterations allowed, and the convergence criterion for the first DNEB run.

3. For optimising the DNEB images using L-BFGS and obtaining transition state guesses

    - MAXBFGS specifies the maximum step length.
    - BFGSSTEPS is used to define the maximum number of steps.
    - The first argument of MAXERISE specifies the threshold energy. Any step that leads to an energy rise above this threshold will be rejected.
    - BFGSMIN designates the convergence criterion on RMS gradient.

4. For obtaining a converged transition state from a candidate geometry using Rayleigh-Ritz minimisation and hybrid eigenvector-following
   - The steps taken during Rayleigh-Ritz minimisation are checked using the trust radius given by TRAD
   - The second argument of MAXERISE specifies the threshold energy. Any step that causes an energy rise above this threshold is rejected.
   - MAXMAX defines the maximum step size that can be taken in uphill direction during eigenvector-following.
   - STEPS is used to specify the maximum number of steps allowed during transition state search using eigenvector-following.
   - The different arguments of the keyword BFGSTS are employed when L-BFGS is used for energy minimisation in the orthogonal subspace during HEF.
   - The geometry and energy of transition state is further checked using NOCISTRANS, LPERMDIST, GEOMDIFFTOL and EDIFFTOL.
5. For connecting a transition state to two local minima
   - MAXSTEP defines the maximum step length that can be taken along the direction of negative eigenmode.
   - PUSHOPT is used to specify the magnitude of individual steps calculated using Golden-section search, maximum iterations allowed, and convergence criterion.
   - The minima obtained are checked for energy and geometry using EDIFFTOL, GEOMDIFFTOL, NOCISTRANS and LPERMDIST.
6. For choosing minima for subsequent newconnect cycles
   - DIJKSTRA EXP is used to choose appropriate minima to obtain the shortest path between reactant and product minima.

7. A new connection cycle is attempted on the chosen minima using NEWCONNECT keyword. The various arguments specified with this keyword are described below.

    - Total number of newconnect cycles allowed to find a connected pathway between the two endpoints
    - The maximum connection attempts between any two minima chosen by DIJKSTRA
    - Initial image density
    - Iteration density
    - The maximum image density
    - The maximum increase in image density for subsequent iteration
    - RMS convergence criterion

The AMBER force field[46] was used with the following parameters

- imin=1 for energy minimization
- ncyc=1 and maxcyc=1 specifies the maximum number of minimization cycle (1 here).
- igb=2 specifies the use of the Onufriev, Bashford and Case generalised Born implicit solvent model, which is the recommended solvent model for nucleic acids.[47] Although new solvent models have been introduced within AMBER they have been parameterised for proteins only.
- ntb=0 defines the non-periodic boundary conditions during the calculation of non-bonded interactions. This setting is recommended when using igb=2.
- rgbmax=25.0 defines that the atoms within 25 Angstroms will be considered as atomic pairs and will be used during the effective Born radii calculation.
- saltcon=0.1 specifies that the concentration of 1-1 (monovalent) mobile ions in the implicit solvent is 0.1 molar. The inclusion of these ions leads to the screening of electrostatic interactions.[48]

- cut=999 specifies the maximum distance in Angstroms that will be used while finding non-bonded (electrostatic and van der Waals) interactions.

The CPPTRAJ[49] script used for calculation of CPDb dihedral in the DNA duplex.

```
dihedral adenine :7@N9,C8,N7,C5,C6,O6,N1,C2,N2,N3,C4,:18@N1,C6,C5,C4,N4,N3,C2,O2,\
:5@N9,C8,N7,C5,C6,O6,N1,C2,N2,N3,C4,:20@N1,C6,C5,C4,N4,N3,C2,O2 \
:6@O3',:7@P,O1P,O2P,O5' \
:5@O3',:6@P,O1P,O2P,O5' \
:6@N7,C5,C4,N9,C8 \
out outdihedA mass
dihedral guanine :8@N9,C8,N7,C5,C6,N6,N1,C2,N3,C4,:17@N1,C6,C5,C7,C4,O4,N3,C2,O2,\
:6@N9,C8,N7,C5,C6,N6,N1,C2,N3,C4,:19@N1,C6,C5,C7,C4,O4,N3,C2,O2 \
:7@O3',:8@P,O1P,O2P,O5' \
:6@O3',:7@P,O1P,O2P,O5' \
:7@N7,C5,C4,N9,C8 \
out outdihedG mass \
dihedral cytosine :8@N9,C8,N7,C5,C6,N6,N1,C2,N3,C4,:17@N1,C6,C5,C7,C4,O4,N3,C2,O2,\
:6@N9,C8,N7,C5,C6,N6,N1,C2,N3,C4,:19@N1,C6,C5,C7,C4,O4,N3,C2,O2 \
:18@O3',:19@P,O1P,O2P,O5' \
:17@O3',:18@P,O1P,O2P,O5' \
:18@N1,C6,C5,C4,N3,C2 \
out outdihedC mass
dihedral thymine :7@N9,C8,N7,C5,C6,O6,N1,C2,N2,N3,C4,:18@N1,C6,C5,C4,N4,N3,C2,O2,\
:5@N9,C8,N7,C5,C6,O6,N1,C2,N2,N3,C4,:20@N1,C6,C5,C4,N4,N3,C2,O2 \
:19@O3',:20@P,O1P,O2P,O5' \
:18@O3',:19@P,O1P,O2P,O5' \
:19@C6,C5,C4,N3,C2,N1 \
out outdihedT mass
```

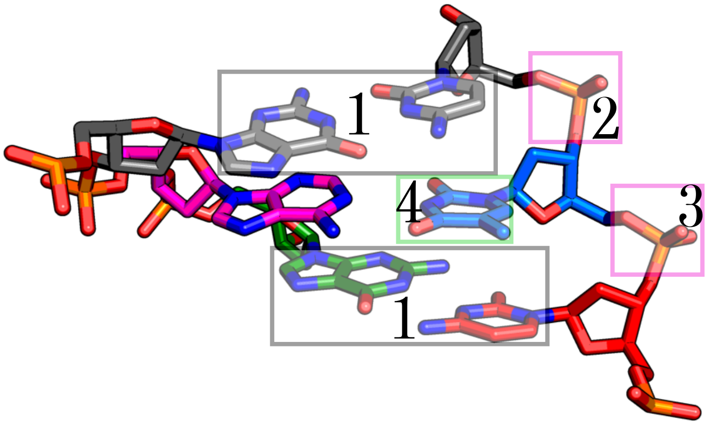

Figure S1: Centre-of-mass pseudo-dihedral angle CPDb as defined by Song *et al.*[50] The four sets of atoms considered under CPDb are the two base-pairs flanking the base of interest (denoted by 1 in the figure), the phosphate group on the 3′ side of the flipping base (denoted by 2 in the figure), the phosphate group on the 5′ side of the flipping base (denoted by 3 in the figure), and the ring atoms of the flipping base (denoted by 4 in the figure). It is important to note that in the case of purine flipping, only atoms of the five-membered ring are considered for the fourth point in the dihedral.

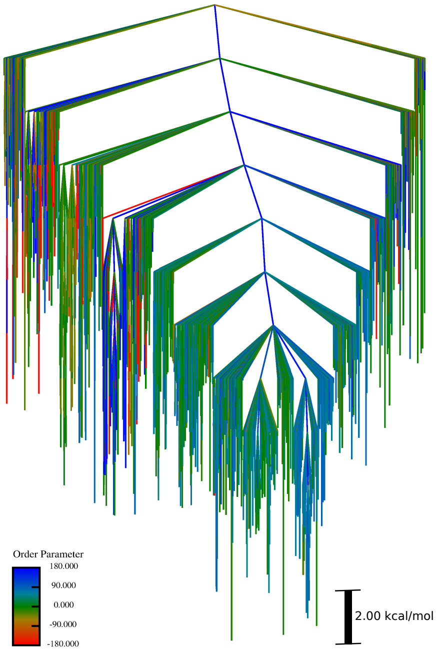

(a)

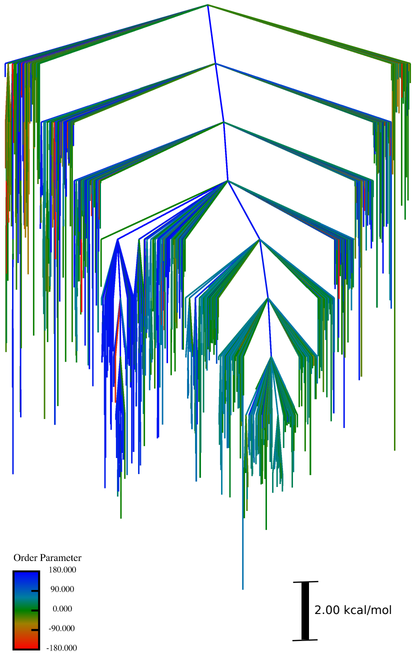

(b)

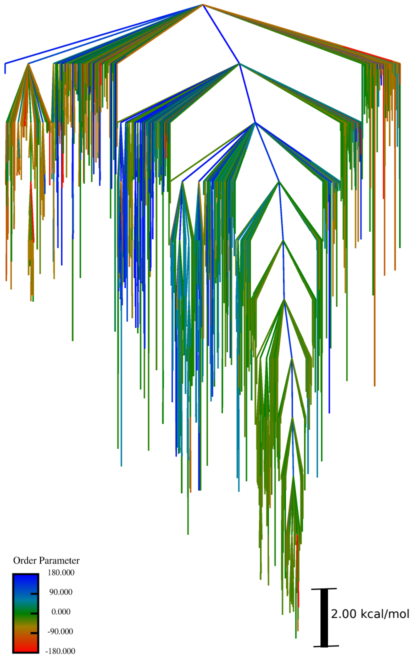

(c)

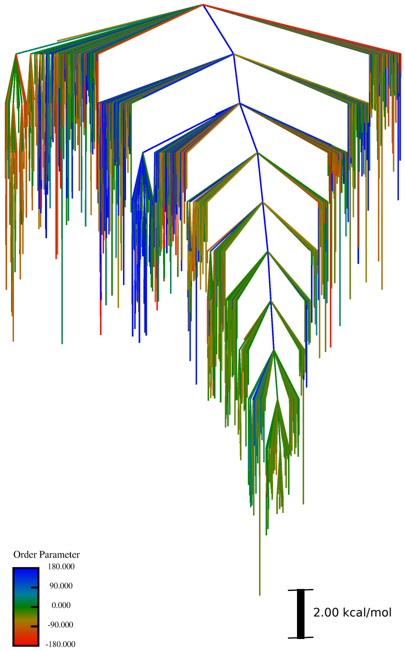

(d)

Figure S2: Free energy disconnectivity graphs for (a) adenine, (b) thymine, (c) guanine and (d) cytosine bases flipped out of a DNA duplex at 300 K. These graphs are the same as those given the main text and the order parameter represents the CPDb dihedral angle values. Here, these graphs are recolored to distinguish between bases flipped out via major (negative CPDb dihedral angle) and minor groove (positive CPDb dihedral angle) pathway.

References

(1) Murrell, J. N.; Laidler, K. J. Symmetries of activated complexes. *Trans. Faraday Soc.* **1968**, *64*, 371–377.

(2) Wales, D. J. *Energy landscapes: Applications to clusters, biomolecules and glasses*; Cambridge University Press, Cambridge, U.K., 2004.

(3) Wales, D. J. Discrete path sampling. *Mol. Phys.* **2002**, *100*, 3285–3305.

(4) Wales, D. J. OPTIM: A program for optimising geometries and calculating pathways. http://www-wales.ch.cam.ac.uk/software.html.

(5) Liu, D. C.; Nocedal, J. On the limited memory BFGS method for large scale optimization. *Math. Program. Ser. B* **1989**, *45*, 503–528.

(6) Henkelman, G.; Jónsson, H. Improved tangent estimate in the nudged elastic band method for finding minimum energy paths and saddle points. *J. Chem. Phys.* **2000**, *113*, 9978–9985.

(7) Henkelman, G.; Uberuaga, B. P.; Jónsson, H. Climbing image nudged elastic band method for finding saddle points and minimum energy paths. *J. Chem. Phys.* **2000**, *113*, 9901–9904.

(8) Trygubenko, S. A.; Wales, D. J. A doubly nudged elastic band method for finding transition states. *J. Chem. Phys.* **2004**, *120*, 2082–2094.

(9) Munro, L. J.; Wales, D. J. Defect migration in crystalline silicon. *Phys. Rev. B - Condens. Matter Mater. Phys.* **1999**, *59*, 3969–3980.

(10) Kumeda, Y.; Wales, D. J.; Munro, L. J. Transition states and rearrangement mechanisms from hybrid eigenvector-following and density functional theory. Application to C10H10 and defect migration in crystalline silicon. *Chem. Phys. Lett.* **2001**, *341*, 185–194.

(11) Dijkstra, E. W. A note on two problems in connexion with graphs. *Numer. Math.* **1959**, *1*, 269–271.

(12) Carr, J. M.; Trygubenko, S. A.; Wales, D. J. Finding pathways between distant local minima. *J. Chem. Phys.* **2005**, *122*, 234903.

(13) Wales, D. J. PATHSAMPLE: A program for generating connected stationary point databases and extracting global kinetics. http://www-wales.ch.cam.ac.uk/software.html.

(14) Strodel, B.; Whittleston, C. S.; Wales, D. J. Thermodynamics and kinetics of aggregation for the GNNQQNY peptide. *J. Am. Chem. Soc.* **2007**, *129*, 16005–16014.

(15) Evans, D. A.; Wales, D. J. Folding of the GB1 hairpin peptide from discrete path sampling. *J. Chem. Phys.* **2004**, *121*, 1080–1090.

(16) Carr, J. M.; Wales, D. J. Folding pathways and rates for the three-stranded β-sheet peptide Beta3s using discrete path sampling. *J. Phys. Chem. B* **2008**, *112*, 8760–8769.

(17) Kleinjung, J.; Fraternali, F. Design and application of implicit solvent models in biomolecular simulations. *Curr. Opin. Struct. Biol.* **2014**, *25*, 126–134.

(18) Tsui, V.; Case, D. Molecular dynamics simulations of nucleic acids with a generalized Born solvation model. *J. Am. Chem. Soc.* **2000**, *122*, 2489–2498.

(19) Chakraborty, D.; Collepardo-Guevara, R.; Wales, D. Energy landscapes, folding mechanisms, and kinetics of RNA tetraloop hairpins. *J. Am. Chem. Soc.* **2014**, *136*, 18052–18061.

(20) Chocholoušová, J.; Feig, M. Implicit solvent simulations of DNA and DNA- protein complexes: agreement with explicit solvent vs experiment. *J. Phys. Chem. B* **2006**, *110*, 17240–17251.

(21) Chakraborty, D.; Wales, D. J. Energy landscape and pathways for transitions between Watson–Crick and Hoogsteen base pairing in DNA. *J. Phys. Chem. Lett.* **2018**, *9*, 229–241.

(22) Onufriev, A. V.; Case, D. A. Generalized Born implicit solvent models for biomolecules. *Ann. Rev. Biophys.* **2019**, *48*, 275–296.

(23) Anandakrishnan, R.; Drozdetski, A.; Walker, R. C.; Onufriev, A. V. Speed of conformational change: comparing explicit and implicit solvent molecular dynamics simulations. *Biophys. J.* **2015**, *108*, 1153–1164.

(24) Gaillard, T.; Case, D. A. Evaluation of DNA force fields in implicit solvation. *J. Chem. Theory Comput.* **2011**, *7*, 3181–3198.

(25) Chakraborty, D.; Wales, D. J. Dynamics of an adenine-adenine RNA conformational switch from discrete path sampling. *J. Chem. Phys.* **2019**, *150*, 125101.

(26) McQuarrie, D. A. *Statistical mechanics*; University Science Books, 2000.

(27) Fejer, S. Self-assembly in complex systems. Ph.D. thesis, University of Cambridge, UK, 2009.

(28) Chakraborty, D. Energy landscapes of nucleic acids. Ph.D. thesis, University of Cambridge, UK., 2016.

(29) Mochizuki, K.; Whittleston, C. S.; Somani, S.; Kusumaatmaja, H.; Wales, D. J. A conformational factorisation approach for estimating the binding free energies of macromolecules. *Phys. Chem. Chem. Phys.* **2014**, *16*, 2842–2853.

(30) Evans, D. A.; Wales, D. J. Free energy landscapes of model peptides and proteins. *J. Chem. Phys.* **2003**, *118*, 3891–3897.

(31) Joseph, J. A.; Röder, K.; Chakraborty, D.; Mantell, R. G.; Wales, D. J. Exploring biomolecular energy landscapes. *Chem. Commun.* **2017**, *53*, 6974–6988.

(32) Strodel, B.; Wales, D. J. Free energy surfaces from an extended harmonic superposition approach and kinetics for alanine dipeptide. *Chem. Phys. Lett.* **2008**, *466*, 105–115.

(33) Rice, O. K.; Ramsperger, H. C. Theories of unimolecular gas reactions at low pressures. *J. Am. Chem. Soc.* **1927**, *49*, 1617–1629.

(34) Rice, O. K.; Ramsperger, H. C. Theories of unimolecular gas reactions at low pressures. II. *J. Am. Chem. Soc.* **1928**, *50*, 617–620.

(35) Wynne-Jones, W. F.; Eyeing, H. The absolute rate of reactions in condensed phases. *J. Chem. Phys.* **1935**, *3*, 492–502.

(36) Evans, M. G.; Polanyi, M. Some applications of the transition state method to the calculation of reaction velocities, especially in solution. *Trans. Faraday Soc.* **1935**, *31*, 875–894.

(37) Eyring, H. The activated complex in chemical reactions. *J. Chem. Phys.* **1935**, *3*, 107–115.

(38) Fain, B. Theory of rate constants: Master equation approach. *J. Stat. Phys.* **1981**, *25*, 475–489.

(39) Frankcombe, T. J.; Smith, S. C. Solving the unimolecular master equation with a weighted subspace projection method. *J. Comput. Chem.* **2000**, *21*, 592–606.

(40) Wales, D. J. Energy landscapes: Calculating pathways and rates. *Int. Rev. Phys. Chem.* **2006**, *25*, 237–282.

(41) Wales, D. J. Calculating rate constants and committor probabilities for transition networks by graph transformation. *J. Chem. Phys.* **2009**, *130*, 204111.

(42) Carr, J. M.; Wales, D. J. Refined kinetic transition networks for the GB1 hairpin peptide. *Phys. Chem. Chem. Phys.* **2009**, *11*, 3341–3354.

(43) Trygubenko, S. A.; Wales, D. J. Kinetic analysis of discrete path sampling stationary point databases. *Mol. Phys.* **2006**, *104*, 1497–1507.

(44) Swinburne, T. D.; Wales, D. J. Defining, calculating, and converging observables of a kinetic transition network. *J. Chem. Theory Comput.* **2020**, *16*, 2661–2679.

(45) Griffiths, M.; Niblett, S. P.; Wales, D. J. Optimal alignment of structures for finite and periodic systems. *J. Chem. Theory Comput.* **2017**, *13*, 4914–4931.

(46) Case, D. A.; Walker, R. C.; Cheatham, T. E.; Simmerling, C.; Roitberg, A.; Merz, K. M.; Luo, R.; Darden, T. Amber 18. 2018; http://ambermd.org/doc12/Amber18.pdf.

(47) Onufriev, A.; Bashford, D.; Case, D. A. Modification of the generalized born model suitable for macromolecules. *J. Phys. Chem. B* **2000**, *104*, 3712–3720.

(48) Srinivasan, J.; Trevathan, M. W.; Beroza, P.; Case, D. A. Application of a pairwise generalized Born model to proteins and nucleic acids: Inclusion of salt effects. *Theor. Chem. Acc.* **1999**, *101*, 426–434.

(49) Roe, D. R.; Cheatham, T. E. PTRAJ and CPPTRAJ: Software for processing and analysis of molecular dynamics trajectory data. *J. Chem. Theory Comput.* **2013**, *9*, 3084–3095.

(50) Song, K.; Campbell, A. J.; Bergonzo, C.; de los Santos, C.; Grollman, A. P.; Simmerling, C. An improved reaction coordinate for nucleic acid base flipping studies. *J. Chem. Theory Comput.* **2009**, *5*, 3105–3113.
